# Synthesis and Modeling of Poly(L-lactic acid) via Polycondensation of L-Lactic Acid

**DOI:** 10.3390/polym15234569

**Published:** 2023-11-29

**Authors:** Alexis Theodorou, Vasilios Raptis, Chrissie Isabella Maria Baltzaki, Thrassyvoulos Manios, Vagelis Harmandaris, Kelly Velonia

**Affiliations:** 1 Department of Materials Science and Technology, University of Crete, 70013 Heraklion, Greece; a-theodorou@materials.uoc.gr (A.T.); chrissie@materials.uoc.gr (C.I.M.B.); 2 Institute of Applied and Computational Mathematics, Foundation for Research and Technology-Hellas, 70013 Heraklion, Greece; v.raptis@external.euc.ac.cy; 3 Department of Computer Science and Engineering, European University Cyprus, 6 Diogenis Str., 2404 Nicosia, Cyprus; 4 Department of Agriculture, Hellenic Mediterranean University, 71410 Heraklion, Greece; tmanios@hmu.gr; 5 Department of Mathematics and Applied Mathematics, University of Crete, 70013 Heraklion, Greece; 6 Computation-Based Science and Technology Research Center, The Cyprus Institute, 2121 Nicosia, Cyprus

**Keywords:** poly(L-lactic acid), lactic acid, polycondensation, polymerization kinetics, bioplastics

## Abstract

We present synthetic experiments of lactic acid (LA) polycondensation to produce poly(lactic acid) (PLA) as well as kinetic modeling calculations that capture the polymer molecular weight increase with time, given the initial concentrations. Tin-octoate-catalyzed polycondensation of (D,L)- or L-lactic acid was carried out in pre-dried toluene after azeotropic dehydration for 48–120 h at 130–137 °C. The polymerization was optimized by varying lactic acid and catalyst concentrations as well as the temperature. Gel permeation chromatography was used to experimentally follow the evolution of molecular weights and the products were characterized by NMR, TGA, DSC and IR. Under optimal conditions, PLLA with weight-average molecular weight (*M*_w_) of 161 kDa could be obtained. The rate equations that describe polycondensation kinetics were recast in a condensed form that allowed very fast numerical solution and calculation of the number-average molecular weight with time. Deviations with respect to the experiment were minimized in a least-squares fashion to determine rate constants. The optimized kinetics parameters are shown to reproduce the experimental data accurately.

## 1. Introduction

Plastic pollution has emerged as a major threat to the environment and public health. It has been shown that out of all plastic ever manufactured globally, a mere 6.1% has been recycled, another 8.4% has been incinerated, 30.1% remains in use and a majority of 55.4% has been disposed of into the environment. With the lifetime of plastic ranging between a few years and several centuries, the above situation has resulted in a waste crisis intertwined with other major challenges such as the depletion of natural resources because of oil consumed to produce plastic, and the carbon footprint associated with such products [1].

The problem of plastic waste has, thus, multifarious aspects calling for a wide range of solutions, such as restrictions imposed on single-use items (or banning them altogether); recycling; reprocessing and incineration; and adoption of degradable materials [2]. The latter can be either fossil-based or biodegradable materials [3] made from renewable biomass capable of chemically or biologically decomposing to produce innocuous compounds. Biodegradable materials certainly have non-negligible lifetimes themselves (otherwise, they would be of no practical use) so they can be part of a viable solution provided they are combined with policies and infrastructure for recycling/reprocessing, composting, and incineration.

Poly(lactic acid), PLA, is a biodegradable polymer typically produced from renewable resources such as corn, wheat, straw or sorghum and more recently, from biowaste [4,5]. PLA can be used as a biocompatible and environment-friendly plastic with a range of applications extending from biomedicine and 3D printing to packaging and other routine daily usages as a result of its physicochemical properties [6,7,8,9,10,11,12,13]. PLA degradation is a result of ester bond hydrolysis which can take place when it is exposed to appropriate environmental conditions. PLA biodegrades into the biofriendly lactic acid or carbon dioxide and water at high temperatures. It degrades slowly [14] but its depolymerization to lactic acid encourages its recycling and composting [15,16,17]. Furthermore, it leaves no trace when incinerated so energy production can be an alternative usage thereof. Overall, PLA significantly reduces the carbon and environmental footprint compared to traditional plastics [18]. Unsurprisingly, the global poly(lactic acid) market was estimated at USD 10^9^ by the end of 2022 and has been projected to reach almost USD 3.1 × 10^9^ by 2032 [19].

PLA is, therefore, an ideal material to consider in the context of local projects based on renewable biomass, waste collection, and recycling, with the twofold aim of adding to the community’s economic welfare and gradually replacing fossil-based non-degradable material. The present work is part of one such project concerning the design and optimization of PLA synthesis from anaerobically digested starch-rich food waste of local hospitality units at the pilot-plant level. 

Depending on the isomer used as starting material, pure L-, D-, or a mixture of isomers, poly(lactic acid) can be produced as poly-L-lactic acid (PLLA), as poly-D-lactic acid (PDLA) or poly-D,L-lactic acid (PDLLA), respectively. The stereochemistry of PLA has an unambiguous impact on its material physicochemical properties [20]. PLA formed by racemic mixtures of lactic acid isomers is an amorphous polymer with a high degradation rate, whereas optically pure PLA is semi-crystalline with a significantly lower degradation rate [21,22].

The most well-established method for the synthesis of optically pure high-molecular-weight PLLA involves ring opening polymerization (ROP) of L-lactides at high temperatures (ca. 200 °C) in the presence of initiators [23], organic solvents [4] and various metal-based catalysts such as tin, zinc, lead, and aluminum [24,25,26]. The efficiency of ROP lies in the fact that no water that could intervene with the polymerization process is produced during the ring-opening process. A mild alternative route is enzymatic ROP, in which free or immobilized lipases are employed as the polymerization catalysts; however, they are suitable only for the synthesis of moderate-molecular-weight PLA (<30 kDa) [27]. Although these procedures are efficient, the synthesis of the single stereoisomer L-lactide from L-lactic acid and the required separation and purification steps have high energy requirements [4]. Therefore, alternative routes for the industrial production of PLLA need to be developed to reduce the environmental footprint of this process.

Direct polycondensation of lactic acid, on the other hand, is a straightforward process, which does not require intermediate lactide synthesis and isolation while the reaction can be induced by azeotropic dehydration of the reaction mixture in solution, or neat by melt polycondensation of lactic acid under high temperatures (above 180 °C) and reduced pressures [28,29]. Major factors associated with polymerization efficiency in this case are the tolerance of the catalyst in water and the efficiency of in situ water removal to minimize water-assisted depolymerization which renders the synthesis of high-molecular-weight PLA challenging [30]. Efficient dehydration of the reaction mixture has been achieved through azeotropic polycondensation of lactic acid in solution in two stages involving initial dehydration of the hygroscopic lactic acid followed by removal of the water produced during the polymerization process. The removal of water in the first step is performed either through azeotropic distillation [31] or through Dean–Stark recycling of the organic solvent [32,33]. During polymerization, continuous dehydration is commonly achieved via solvent recycling through molecular sieves, often by means of reduced pressure [32,33]. The latter approach is highly promising for the large-scale production of PLA due to its projected lower operating costs.

The kinetics of polymerization and PLA formation can be cast in forms of varying complexity, depending on the underlying microscopic mechanism. For instance, (D,L)-lactic acid polymerization with aluminum isopropoxide [34] and L-lactide living polymerization with tin(II) ethyl hexanoate as a catalyst [35] can be described by single first-order linear differential equations for the lactide concentration with time. In these systems, the number-average molecular weight of the product is a linear function of the monomer conversion. On the other hand, Dubey et al. [36] and Dubey et al. [37] propose a three-stage mechanism for the catalytic ring-opening polymerization of lactide (activation, propagation, chain-transfer termination) accompanied by two side reactions, with the corresponding number of rate constants and parameters that should be fitted to experimental data. In this model, all intermediate and final products up to a maximum chain length are explicitly considered, requiring a set of up to thousands of differential equations in order to predict reliable results. In a similar vein, Mehta et al. [38] modeled ring-opening polymerization of lactic acid catalyzed by stannous octoate, assuming an initiation and a propagation stage, as well as two alternative termination steps (transfer to the monomer, cationic mechanism). The model that took all intermediate and final products into account required a number of differential equations proportional to the maximum degree of polymerization under consideration (in their work, 5 × 10^3^). The resultant set was solved numerically.

Finally, Harshe et al. [39,40] adopted a similar approach when studying the polycondensation of lactic acid with continuous water removal, and proposed a model along the lines of Dotson et al. [39] accounting for all intermediate and final products using three rate constants (polymerization, depolymerization, and water removal by diffusion) and assuming no dependence on the size of reacting species. The authors considered a maximum degree of polymerization of 10^3^ and numerically solved a proportionately large set of differential equations. They also considered the limiting cases of closed systems (no water removal) and open systems (complete water removal) that greatly simplified the model and assumed analytical solutions.

The above multi-stage models can predict the whole molecular weight distribution (up to a user-defined upper bound) from which the number- and weight-average molecular masses can be computed, which partly justifies their complexity. On the other hand, in the case of polycondensation, a simpler alternative exists that can provide reliable predictions of number-average molecular weights, as shown later in this article. The present work combines the development of a new PLA catalytic polymerization scheme with the modeling of its kinetics aimed at providing a quick means for the reliable estimation of molecular weight with time when using our synthetic method.

One of the main concerns for the development of PLA production units based on feedstocks or recycled food waste is to operate under mild conditions in order to conform with existing regulations but also keep operating costs low and secure the economic feasibility of the production. To satisfy such requirements, we present herein a study of the polycondensation of lactic acid catalyzed by tin octoate under relatively low temperatures and ambient pressure. The modeling approach that accompanies this study allows for quick estimations of rate constants, which in turn will facilitate future optimization of the process and subsequent scale-up studies. We present the experimental and modeling methodologies. The experimental setup is briefly outlined with full experimental details presented in the electronic Supplementary Materials. The modeling approach is discussed in more detail, using the model of Harshe et al. [40] and its assumptions as a starting point; then, a simplification of the model is carried out and the procedure for determining the rate constants is described. Experimental and modeling results are presented and discussed in Section 3 and our conclusions are summarized in Section 4. The Supplementary Materials list the materials and provide details of the experimental procedures and characterization techniques, and summarize experimental results. Finally, Appendix A contains the notation used in the mathematical analysis presented herein.

## 2. Materials and Methods

### 2.1. Experiments

The experimental setup and synthesis are briefly reviewed here; the reader is referred to the electronic Supplementary Materials for details (2 Materials and 3 Analytical Techniques). Mixtures of commercial D,L- or L-lactic acid and dry toluene were placed in a two-neck round bottom flask equipped with a graduated Dean–Stark receiver and a condenser. The mixture was heated at 140 °C in ambient pressure, water was removed by means of the Dean–Stark receiver, and the reaction mixture was subsequently purged with nitrogen. A solution of the catalyst tin octoate in dry toluene was subsequently added, and the reaction mixture was azeotropically dehydrated by means of a dropping funnel containing molecular sieves for up to 120 h at temperatures ranging from 130 to 137 °C. To monitor the progress of the reaction with time, aliquots (0.5 mL) were carefully withdrawn from the reaction mixture at specific time points. Upon completion of the reaction (120 h), the solvent was removed in vacuo and the residue was dissolved in THF or chloroform, filtered, and analyzed with gel permeation chromatography (GPC). All reported molecular weights were calculated by comparing them with polystyrene standards and therefore the MWs provided relate to polystyrene of the same hydrodynamic volume. The produced polymers were characterized with nuclear magnetic resonance (NMR) and infrared (IR) spectroscopy. Thermal analysis was performed with thermogravimetric analysis (TGA) and differential scanning calorimetry (DSC).

### 2.2. Kinetic Model

Our starting point will be the above-mentioned model of Harshe et al. [39,40] with some adjustments to conform to the experimental setup of our study. We consider the following reversible step growth polymerization mechanism:

(1) $$\mathrm{HOOC}\left(R_{i}\right)\mathrm{OH}+\mathrm{HOOC}\left(R_{j}\right)\mathrm{OH}\begin{array}{c} k_{1}\\ ⇄\\ k_{-1} \end{array}\mathrm{HOOC}\left(R_{i+j}\right)\mathrm{OH}+H_{2}O$$

where R*_i_* and R*_j_* are polymer chains, *i* and *j* denote degrees of polymerization, and the rate constants *k*_1_ and *k*_−1_ correspond to polymerization and depolymerization constants, respectively. To obtain the polycondensation kinetic model, the following assumptions are made:

(a) Constants *k*_1_ and *k*_−1_, are independent of molecular weight, thus reducing the problem size when it comes to the determination of rate constant values that capture the experimental data on polymerization kinetics.
(b) The carboxyl and hydroxyl groups are assumed to be equally reactive; it is also assumed that no autocatalysis occurs, and no side reactions take place.
(c) Moreover, the liquid phase is fully mixed and homogeneous, without temperature or concentration gradients, whereas the volatility of lactic acid and all polymer products is negligible.
(d) Finally, we postulate that water is vaporized and removed from the liquid phase at a rate proportional to the liquid’s water mole fraction, *x*_w_. Briefly, we assume a driving force proportional to the change in water’s partial pressure between the vapor–liquid interface and the solvent reflux point past the water trap. With the complete removal of water by the water trap, the partial pressure at the reflux point will be zero so the driving force will be proportional to the water’s partial pressure at the liquid–vapor interface. If the water mole fraction in the liquid phase varies within a narrow range of small values during the experiment, then we can assume the vapor’s partial pressure proportional to fugacity, thus to liquid water mole fraction, by a more or less constant coefficient, *k*_w_. Under this assumption, the water removal rate becomes independent of the vapor pressure of water and the total pressure of the reactor.

The validity of the arguments justifying the above assumptions, and the extent to which they affect our results, are examined by comparing the model predictions to the experimental data. In particular, the assumption of a small variation of the water mole fraction needs to be revisited at the end of our calculations; for this assumption to hold, the liquid phase’s water content will have to remain low throughout the polymerization because the initial conditions assume a nearly dry solvent–catalyst–lactic acid mixture at the beginning of the reaction.

Given the polymerization mechanism and the model assumptions described above, we can develop constitutive mass balance equations for the (a) formation and consumption of polymer chains, (b) consumption of lactic acid, and (c) water production, consumption, and removal via vaporization. Each species R*_i_* can be produced either by the combination of shorter chains or by the consumption of longer ones combined with water, whereas at the same time, it can be consumed either by breaking down to shorter chains or by reacting with other species to produce longer molecules. In these elementary reactions, all possible ways in which a chain can be broken down or consumed have to be enumerated. Thus, the concentration equation for polymer chains of all chain lengths is written as follows:

(2) $$\frac{dP_{i}}{dt}=k_{1}\sum_{j=1}^{i-1}{P_{i-j}P}_{j}+2k_{-1}W\sum_{j=i+1}^{\infty }P_{j}-{\left(i-1\right)k}_{-1}WP_{i}-2k_{1}P_{i}\sum_{j=1}^{\infty }P_{j}\;$$

where *P_i_* and *W* denote molar concentrations of polymer chains consisting of *i* monomers (1 ≤ *i* < ∞) and water, respectively.

In the above equation, the prefactors of 2 in the second and fourth right-hand-side terms, are explained by the possibility of reacting in two ways: Any R_*i*+*k*_ chain (*k* > 0) can split at two points, either as R*_i_*R*_k_* or as R*_k_*R*_i_* to form a chain of length *i* and a leftover of length *k*, i.e.,

$$\mathrm{HOOC}\left(R_{i}\mathrm{COO}R_{k}\right)\mathrm{OH}+H_{2}O\to \mathrm{HOOC}\left(R_{i}\right)\mathrm{OH}+\mathrm{HOOC}\left(R_{k}\right)\mathrm{OH}$$

or

$$\mathrm{HOOC}\left(R_{k}\mathrm{COO}R_{i}\right)\mathrm{OH}+H_{2}O\to \mathrm{HOOC}\left(R_{k}\right)\mathrm{OH}+\mathrm{HOOC}\left(R_{i}\right)\mathrm{OH}$$

Likewise, R*_i_* can be combined with R*_j_* in two ways, either

$$\mathrm{HOOC}\left(R_{i}\right)\mathrm{OH}+\mathrm{HOOC}\left(R_{j}\right)\mathrm{OH}\to \mathrm{HOOC}\left(R_{i}\mathrm{COO}R_{j}\right)\mathrm{OH}+H_{2}O$$

or

$$\mathrm{HOOC}\left(R_{j}\right)\mathrm{OH}+\mathrm{HOOC}\left(R_{i}\right)\mathrm{OH}\to \mathrm{HOOC}\left(R_{j}\mathrm{COO}R_{i}\right)\mathrm{OH}+H_{2}O$$

The monomer consumption balance can be derived directly from the balance of polymer chains by setting *i* = 1, thus:

(3) $$\frac{dP_{1}}{dt}=2k_{-1}W\sum_{j=2}^{\infty }P_{j}-2k_{1}P_{1}\sum_{j=1}^{\infty }P_{j}\;$$

Finally, to write down the balance for water consumption, we consider (a) water production from polymerization elementary reactions, (b) water removal from depolymerization reactions and (c) water removal due to vaporization:

$$\frac{dW}{dt}=k_{1}\sum_{i=1}^{\infty }P_{i}\sum_{j=1}^{\infty }P_{j}-{\left(i-1\right)k}_{-1}W\sum_{i=1}^{\infty }P_{i}-k_{w}x_{w}$$

or

(4) $$\frac{dW}{dt}=k_{1}{\left(\sum_{i=1}^{\infty }P_{i}\right)}^{2}-{\left(i-1\right)k}_{-1}W\sum_{i=1}^{\infty }P_{i}-k_{w}x_{w}\;$$

where *k*_w_ is the water removal rate due to vaporization and the water mole fraction, *x*_w_, is given by

$$x_{w}=\frac{W}{S+C+\sum_{i=1}^{\infty }P_{i}+W}$$

The *i* − 1 factor of the second right-hand-side term is explained by similar arguments as before; namely, R*_i_* can be broken down to shorter chains like:

$$\mathrm{HOOC}\left(R_{j}\mathrm{COO}R_{i-j}\right)\mathrm{OH}+H_{2}O\to \mathrm{HOOC}\left(R_{j}\right)\mathrm{OH}+\mathrm{HOOC}\left(R_{i-j}\right)\mathrm{OH}$$

where *j* = 1, 2, …, *i* − 1.

Our initial conditions are (a) *W*(0) > 0 (a positive but very small value in the range of 10 ppm or less), (b) *P*_1_(0) > 0 (known initial concentration), and (c) *P_i_*(0) = 0 for every *i* > 1. We assume equal rates of solvent removal and reflux (e.g., no trapping of solvent in water trap) and very small changes in the volume of the liquid phase in the reaction vessel (see also assumption of low water content, above) so that the solvent concentration *S* will remain more or less constant throughout the experiment. The same holds for the catalyst concentration, *C*. Then, the water mole fraction in the liquid phase at the beginning of the reaction equals

(5) $$x_{w}\left(0\right)=\frac{W_{0}}{S+C+P_{1}\left(0\right)+W_{0}}\;\;$$

### 2.3. Simplification

The model presented in the previous section, Equations (2)–(4), can be solved numerically by assuming a realistically large maximum degree of polymerization, of the order of 10^3^, implying a correspondingly large size of the set of equations. To optimize the model so that it reproduces experimental results, a call to the kinetics equation solver has to be reiterated several times over a range of test values of rate and water-removal parameters until these converge to their optimal values. Given the problem size and possible complications due to numerical instabilities and the inherent nonlinearity, calculations can be time-consuming and hard to complete.

Harshe et al. [40] were able to simplify their model in the extreme cases of a closed system (no water removal) and an open system with complete water removal. We proceed to show that it is possible to simplify the same model without special considerations about the water content (to the extent that the assumptions of Section 2.2 hold). Indeed, careful examination will allow us to condense the kinetics model to a set of just three equations capable of yielding predictions of the number-average molecular weight with time. To do so, we write the polymerization reaction as

$$\underset{P}{\underbrace{\mathrm{HOOC}\left(R_{i}\right)}}\underset{A}{\underbrace{\mathrm{OH}}}+\underset{B}{\underbrace{H\text{-}}}\underset{Q}{\underbrace{\mathrm{OOC}\left(R_{j}\right)\mathrm{OH}}}\;\begin{array}{c} k_{1}\\ ⇄\\ k_{-1} \end{array}\;\underset{P}{\underbrace{\mathrm{HOOC}\left(R_{i}\right)}}\underset{Q}{\underbrace{\left(\mathrm{COO}R_{j}\right)\mathrm{OH}}}+\underset{\mathrm{AB}}{\underbrace{H_{2}O}}$$

or

(6) $$P\text{-}A+B\text{-}Q\;\begin{array}{c} k_{1}\\ ⇄\\ k_{-1} \end{array}\;P\text{-}Q+A\text{-}B$$

Then, we can describe the process as a reaction between chemical bonds of HOOCR*i*-OH (P-A) and HOR*j*COO-H (B-Q), in the direction of polymerization, or ester (P-Q) and water’s hydrogen–hydroxyl pair (H-OH or A-B), in the reverse direction. In this process, particular bonds of those types exchange their constituent groups to give new bonds. Consequently, we can ignore the parts of the reacting species that do not participate directly in the reaction, and write rate equations in terms of concentrations of bonds that actually react. To this end, we define *a* = [A-P], *b* = [Q-B], *c* = [P-Q] (meaning the bond that connects the two moieties, e.g., P and Q, not the whole chain) and *w* = [A-B]. Given that A-P and Q-B bonds are actually situated at the two ends of each lactic acid or higher chain molecule, their concentrations should be identical, *b* = *a*. Then, we arrive at the following simplified set of rate equations:

(7) $$\frac{dc}{dt}=k_{1}a^{2}-k_{-1}cw$$

(8) $$\frac{da}{dt}=k_{-1}cw-k_{1}a^{2}=-\frac{dc}{dt}\;$$

and

(9) $$\frac{dw}{dt}=k_{1}a^{2}-k_{-1}cw-k_{w}x_{w}=\frac{dc}{dt}-k_{w}x_{w}\;$$

where

(10) $$x_{w}=\frac{w}{2a+c+w+S+C}$$

The set of Equations (7)–(9) should be considerably easier to solve than Equations (2)–(4). Our initial conditions now read *c*(0) = 0, *a*(0) = *a*_0_ (lactic acid’s initial concentration) and *w*(0) ≈ 0. Then, an estimator of the average molecular weight with time can be given by the expression

(11) $$M_{n}\left(t\right)=\frac{c\left(t\right)}{a\left(t\right)}{FW}_{\mathrm{mono}}$$

where FW_mono_ is the repeat unit’s formal weight. This result comes from the simple fact that the number of P-Q bonds (concentration *c*) that corresponds to a single A-P or Q-B bond (concentration *a*) is merely the degree of polymerization. Given that there is just one bond of A-P type per molecule, the denominator in the above expression counts the molecules whereas the numerator sums their degrees of polymerization. In other words, the above estimator corresponds to the number-average molecular weight. Most notably, the simplified model does not require the definition of an arbitrary upper bound to the degree of polymerization (unlike its predecessor, Equations (2)–(4), which cannot be solved in practice without setting such an upper limit). Of course, there is a price to pay for this simplification, which lies in the model’s inherent inability to predict the molecular weight distribution and the index of polydispersity.

In principle, the end groups of a long enough chain can react with each other to give rise to a ring polymer. Thus, an unknown fraction of the ester bonds present in the system should be subtracted before calculating the number-average molecular weight, *M*_n_, from Equation (11), implying that the estimate based on the total number of P-Q bonds represents an upper bound to *M*_n_. However, for non-negligible fractions of ring polymers to form, very low reactant concentrations are required. The conditions of our experiments are expected to favor the formation of linear products over ring ones, so it is safe to assume that the above estimator, Equation (11), should be a very good approximation to the actual number-average molecular weight of linear PLA.

### 2.4. Numerical Simulations

To proceed with the solution of the above set of ODEs, we need to estimate the rate and water removal constants: *k*_1_, *k*_−1_ and *k*_w_. Given a set of *N* experimentally determined number-average molecular weights versus time *t_i_*, *i* = 1, 2, …, *N*, we seek such rate and water removal constant values that the solution of Equations (7)–(9) yields predictions that minimize the error with respect to the measurements. Here, this was achieved by defining the objective function, *F*.

(12) $$F=\sum_{i=1}^{N}{\left(M_{n}^{\mathrm{num}}\left(t_{i};p\right)-M_{n,i}^{\exp}\right)}^{2}\;\;$$

where the rate constants *k*_1_ and *k*_−1_ and proportionality coefficient *k*_w_ are collectively denoted by p, and the subscript ‘n’ denotes the number average of molecular weight of the *i*-th experimental data point and superscripts ‘exp’ and ‘num’ denote experimentally measured and numerically computed values, respectively. The numerically predicted molecular weights are calculated as functions of time, *t*, by solving the rate equations, Equations (7)–(9), and then applying Equation (11). Thus, the proposed minimization problem can be formulated as

$${min}_{p}\left\{F\right\},p=\left\{k_{1},k_{-1},k_{w}\right\}.$$

We solved the above minimization problem with the aid of the derivative-free Nelder–Mead minimization algorithm (Simplex) [41] as implemented in the Merlin multidimensional optimization package [42]. During each call to the minimization routine, the rate equations were solved using the current values of the rate and water removal constants, and the objective function, *F*, was computed by comparing each experimental point with the value computed during the timestep coinciding with the corresponding experimental time.

The rate equations were solved by a simple Euler method with a timestep of 1 s for a time span of 200 h (720,000 s). Initial conditions were set according to the initial concentrations calculated as shown in the example of Table S1 (the values therein represent an estimated scenario as explained in the Results section, Section 3.2). Despite the small timestep, as the rate and water removal constants were altered by the minimization routine, instabilities would occasionally arise due to large absolute values of the computed derivatives. This issue was addressed with the aid of an auto-adaptive variable step scheme that worked as follows: First, it was determined that an acceptable change, Δ*x*, from the current value *x*(*t*) of a given variable *x* to its updated value *x*(*t* + *h*) where *h* denotes the variable step, should not exceed a certain fractional threshold (set to 10%) of the old value. Thus, *h* would be reset to a smaller value and a new updated value would be calculated until achieving the desired, smaller than our threshold, change rate, Δ*x*. On the other hand, a compromise was made by imposing a lower bound of 10^−10^ to *h* to avoid extremely small steps that would cause the solver to stall. It was shown in practice that this constraint to the range of *h* values had only a minimal effect on the solutions in the form of the values occasionally ‘jittering’ around their main trend line. The user-defined timestep, Δ*t*, that was set to 1 s served as an upper bound to *h*.

Overall, the solver performed very well, taking fractions of a second to integrate the rate equations within the desired time span on an ordinary laptop computer. When calling the solver through the optimizer, an additional safety measure was taken by penalizing the objective function, *F*, by a large multiplier, in the case of unstable solutions (e.g., values tending to infinity). A number of initial values were tried for the rate and water removal constants until attaining an optimal solution that exhibited good reproduction of our experimental measurements.

## 3. Results and Discussion

### 3.1. Experiment

Lactic acid is a highly hygroscopic compound; hence, it is usually handled in concentrated aqueous solutions (10–40% by weight) [43]. In our experiments, commercial (L or DL) lactic acid containing about 12% residual water was used as starting material. Most of the residual water was collected in a Dean–Stark receiver and removed (i.e., 1.2 g water per 8.1 g of commercial lactic acid) along with toluene during an azeotropic distillation. During this process, 5% *w*/*w* of lactic acid was converted to L-lactide as determined by ^1^H-NMR (Figure S1).

The polymerization process was performed in a second step during which the Dean–Stark apparatus was replaced by a glass tube packed with activated molecular sieves 3 Å and the catalyst tin octoate catalyst was added. The molecular-sieve-packed tube was used to remove both residual water and water produced during polymerization as it was previously shown that recycling of the solvent through the molecular sieves may reduce the water content of the mixture to as low as 3 ppm or less [21,32]. Additionally, the temperature was slightly reduced (130–137 °C) during polymerization to limit further formation of lactides, thus avoiding loss of stereochemistry. Lactides are commonly formed at temperatures of 140 °C or higher [4,44], while their polymerization occurs above 180 °C under reduced pressure [4]. Even though formation of lactides is unlikely to occur under the comparatively milder conditions of the protocol reported herein, the prolonged reaction times might lead to partial reaction of any lactides formed during the first step. In such a case, the optical purity of the resulting PLA would not be guaranteed, since a mixture of D-, L- and meso-Lactides could form as a result of the racemization of L-lactic acid. Targeting high-molecular-weight PLAs, the polycondensation was allowed to proceed for specified amounts of time after which the product was dissolved in THF or chloroform, filtered and studied with ^1^H-NMR and GPC.

**Figure 1:**
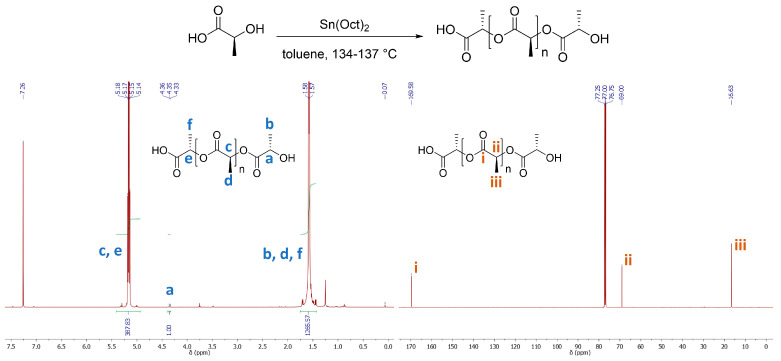
^1^H-NMR (left) and ^13^C-NMR (right) of the PLA in CDCl_3_ formed by the polycondensation of L-lactic acid. Reaction conditions: L-lactic 58.4 wt.%, 0.56 wt.% catalyst, polymerization at temperatures between 134 and 137 °C (Table S2, Entry 2).

In the ^1^H-NMR spectrum of PLLA (Figure 1 and Figure S2), the double peak at 1.52 ppm is attributed to the methyl groups of the main polymeric chain (Figure 1 and Figure S2, protons c) and the quartet at 5.10 ppm to the adjacent methine protons (Figure 1 and Figure S2, protons a). In the ^13^C-NMR spectrum of PLLA, three peaks appear at 169.6, 69.0 and 16.6 ppm which can be attributed to the ester carbonyl, methines and methyl groups of the main polymeric chain, respectively (Figure 1 and Figure S3). To conclude whether the direct polycondensation of L-lactic acid led to the formation of optical pure PLLA, the reaction was also performed using the racemic mixture of DL-lactic acid. As shown in the spectra acquired for PDLLA (Figures S4 and S5), the presence of diastereomers causes an easily detectable change in the multiplicity of the characteristic peaks of the polymer. Hence, the NMR spectra of PLA provided a first indication that the direct polycondensation of L-lactic acid at the reported reaction conditions led to the formation of optically pure PLLA. Notably, no formation of terminal alkenes could be detected in either the ^1^H- or ^13^C-NMR spectra of the products (Figure 1, Figures S2 and S3), which could be an indication of thermal degradation of PLLA [45]. 

The polycondensation reaction was optimized in terms of temperature, monomer and catalyst concentration and reaction time (Table S2). Reaction times prolonged by as much as 5 days were found to be important for the synthesis of high-molecular-weight PLLA. In particular, the average molecular weight (*M*_n_) of PLA was 41 kDa (Table S2, Entry 1) after 92 h while it almost doubled (81 kDa) when the reaction mixture was further heated for 28 h (Table S2, Entry 2). Entry 2 in Table S2 provides the optimal polycondensation conditions identified in this study based on the targeted increased PLA molecular weights. Alterations either in concentration or temperature led to the formation of lower-molecular-weight PLAs (Table S2, Entries 3–5 and 8, respectively). The importance of dehydration was clearly demonstrated when the polycondensation of DL-Lactic acid was performed without stirring and/or solvent recycling, yielding only low-molecular-weight products under otherwise the same reaction conditions (Figure S6, Table S2, Entries 9 and 10). Aiming to synthesize high-molecular-weight PLLA without prolonged reaction times, we assessed the cross-linker 1,4-phenylene diisocyanate. The cross-linker was found to provide a fast (20 min) and efficient means to increase the molecular weight of PLLA (i.e., from 43 kDa to 96 kDa within 20 min, Table S2, Entry 6). However, further heating of the reaction mixture in the presence of the cross-linker (from 96 kDa to 70 kDa after 48 h, Table S2, Entry 7), led to a molecular weight decrease (Figure S7), which is attributed to depolymerization of PLLA by either cleaving main polymer chain ester bonds [46] and/or the amide bonds formed after the addition of the cross-linker [47].

**Figure 2:**
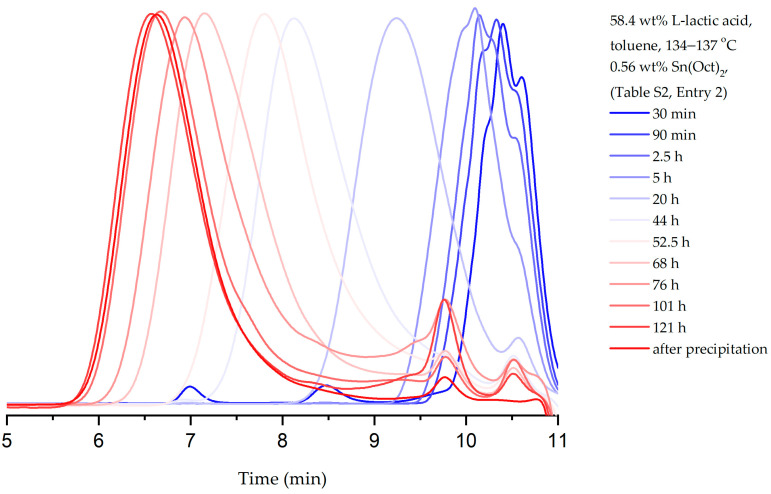
Monitoring PLLA molecular weight increase with time by GPC under optimal polymerization conditions (Table S2, Entry 2). GPC graphs of aliquots (0.5 mL) withdrawn from the reaction mixture at specific time points during polymerization and after isolation.

To get insight into the course of polycondensation and provide data for modeling, the progress of lactic acid polycondensation was monitored with GPC at the optimum conditions (Figure 2 and Figure S8, Table S2, Entry 2). In the initial stages of polycondensation (20 h), oligomers with a degree of polymerization (DP) as low as 10 (*M*_n_ = 947) and dispersity (Đ) at 1.52 were detected. The formation of low-molecular-weight PLLA (*M*_n_ = 5766 kDa) was detected after 52.5 h total polycondensation time (Đ = 2.40). Further heating (68 h) led to a significant increase in the average-molecular-weight PLLA (23 kDa, Đ = 2.15). The polymerization was terminated after 120 h, providing high-molecular-weight PLLA (*M*_n_ = 81 kDa, *M*_w_ = 149 kDa and Đ = 1.84).

FT-IR spectroscopy revealed all the characteristic vibrations of PLLA, [24,48,49,50] i.e., the characteristic stretch of the C-H bonds of polymer methines at about 3000 and 2950 cm^−1^ along with their deformation vibrations around 1380 and 1360 cm^−1^, and the strong stretching and bending vibration of ester carbonyl groups at ca. 1755 and 1265 cm^−1^, respectively, accompanied by the C-O stretching vibrations between 1080 and 1230 cm^−1^ (Table S3). Notably, terminal alkene C-H vibrations of terminal alkenes which could be a result of thermal degradation of PLLA were not detected in any case, supporting the findings of NMR spectroscopy [45]. As shown in Figure S9, the IR spectra of moderate and high *M*_w_ PLLA were found to be almost identical (Table S2, Entries 1 and 2, respectively), supporting the chromatographic data indicating the formation of oligomers at the early stages of polymerization. Additionally, the absorption peak at 1525.7 cm^−1^ in the IR spectrum of PLLA synthesized in the presence of the cross-linker (Table S2, Entry 7) was attributed to the N-H bending vibration of the amide bond formed after chain extension.

The thermal behavior of three PLLA samples selected on the basis of their molecular weight, i.e., low-, medium- and high-*M*_n_ PLLA (41, 70 and 81 kDa, Table S2, Entries 1, 7 and 2, respectively) is shown in the thermogravimetric (TGA) and derivative mass loss (dTG) curves presented in Figure S10. A small weight loss of about 0.5 wt.% seen in all samples below 100 °C can most probably be attributed to the evaporation of residual water. A single thermal decomposition step starting at around 170 °C and ending at 280 °C was seen on the TGA of both the low- and medium-molecular weight PLLA. TGA showed two decomposition steps with no clear boundary for the high-molecular-weight PLLA which was synthesized under optimal conditions (Table S2, Entry 2) with the prominent step at ca. 276.1 °C and a secondary one at 258.2 °C, most probably stemming from the presence of a minute amount of lower-molecular-weight PLLA in the final product as also seen in GPC analyses (Figure S8). The decomposition temperature of all PLLAs at 5% and 50% mass loss (T_0.05_ and T_0.50_, respectively), as well as their maximum decomposition temperature (T_d,max_) and rate were recorded (Figure S10). The temperatures corresponding to 5% and 50% mass loss were found to be 236.9 °C and 268.4 °C for the high-*M*_n_ polymer while the respective temperatures for the lower-*M*_n_ PLAs were found to be 15–20 °C less. The maximum decomposition temperature (T_d,max_) for the high-*M*_n_ polymer was 296.5 °C, for the low-*M*_n_ PLA was 278.1 °C and for the medium-*M*_n_ PLA was 283.7 °C. The maximum rate of the main decomposition step for the high-*M*_n_ polymer was determined with dTG analysis at 275.7 °C while for the medium- and low-molecular-weight PLLAs it was at 261.6 °C and 256.2 °C, respectively (Figure S10).

Differential scanning calorimetry (DSC) measurements of the annealed PLLA samples (Table S2, Entries 1, 2 and 7) are presented in Figure S11. The first thermal-cooling cycle was performed to reduce the thermal history of the samples; therefore, the data collected by this process were not evaluated (Figure S10 left, Table S4) [51,52]. The glass transition temperature (T_g_) of the medium-*M*_n_ PLLA (41 kDa) was 59.9 °C, 5.3 °C less than the T_g_ of high-*M*_n_ PLLA (81 kDa, Table S4, Entries 1 and 2). The T_g_ of the PLLA synthesized in the presence of the cross-linker (medium-*M*_n_) was calculated to be 61.1 °C (70 kDa, Table S4, Entry 7). The polymer with the high-*M*_n_ (Table S4, Entry 2) was found to exhibit the highest cold crystallization temperature (T_cc_, 129.1 °C) and the lowest degree of crystallinity (16.9%), in agreement with what has been previously reported in the literature [53]. The degree of crystallinity was calculated using Equation (13) [54]:

(13) $$C\%=\frac{∆H_{m}}{{∆H}_{m}^{o}}$$

Interestingly, the DSC graphs provided two melting peaks for the polymers with medium molecular weight (40 and 71 kDa, Table S2 Entries 1, 7), while a single melting point value was found for the higher-molecular-weight PLLA (81 kDa, Table S2, Entry 2). The double melting behavior of PLA can be related with the formation of different crystal structures, known as α-form melting at higher temperature, and β-form melting at relatively lower temperatures [55,56,57]. 

### 3.2. Modeling

Initial concentrations were determined according to the data shown in Table S1. The initial mass of dry LA was estimated according to the following reasoning. Removal of residual water (0.93 g confirmed by ^1^H-NMR) leaves 7.11 g of dry LA. However, this is actually an upper bound because of further conversion to lactide (by 5% wt.) and oligolactic acid (of known mass evidenced by ^1^H-NMR). Taking the conversion to lactide into account, we obtain an intermediate estimated dry LA mass of 6.75 g, under the assumptions that lactide does not react under the conditions of our experiments and no conversion to oligomers has taken place. To determine a lower bound that takes the early formation of oligolactic acid into account, we account for its own share of reactive bonds that will be included as an appropriate correction. We consider an unknown average degree of polymerization, *n*_p_, of the molecules formed according to the reaction

*n*_p_ LA → (LA)*n*_p_ − (*n*_p_ − 1) H_2_O

The material balance allows to determine an effective number of LA moles (i.e., ones that have the same number of reactive bonds as the actual mixture of LA and oligolactic acid) in the spirit of our simplified kinetic model. This is equal to *n*_LA,0_–*n*_w_ where n_LA,0_ is the number of LA moles prior to the early formation of its oligomers, and *n*_w_ are the moles of water formed (the same reasoning is actually applicable to any ensemble of reactions for all possible values of *n*_p_). By subtracting the residual water from the 1.2 g total water removed during the initial stage of the experiment, and further considering a small amount of water produced during the partial conversion to lactide (0.0355 g), we end up with 0.2345 g of water produced by oligomerization. Then, we can find the estimated effective initial moles, and finally the corresponding estimated initial mass of dry LA (lower bound) equal to 5.58105 g. 

**Figure 3:**
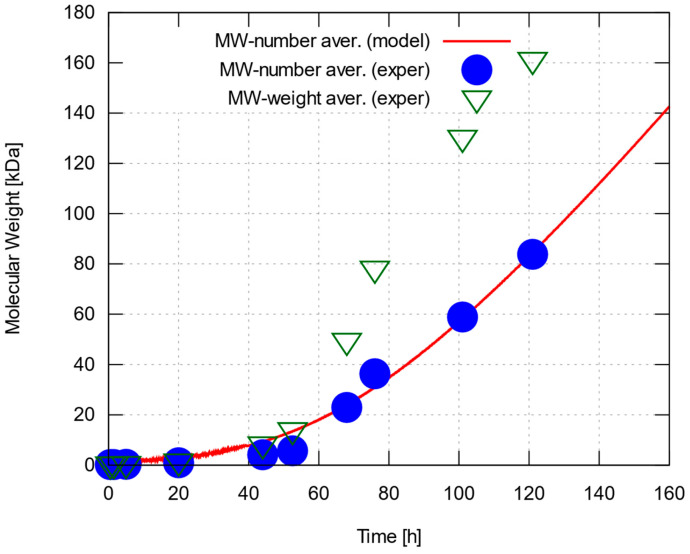
Number and weight-average molecular weight of PLLA synthesized under optimal conditions, and corresponding predictions by kinetic model after optimizing rate and water removal constants to match experimental data. Results shown for the upper bound of estimated initial dry LA mass, 7.11 g (almost identical results were obtained for the other two estimated values of LA mass; see main text for details).

Our calculations based on the measurements taken under optimal experimental conditions for all three estimated dry LA masses, as above, resulted in the rate and water removal constants summarized in Table 1. The displayed figures represent a proposed range of values that accounts for the uncertainty of the initial dry LA mass associated with the side reactions at the beginning of the experiment. The predictions vary by about 6% to 12% depending on LA’s initial mass, which by comparison, was allowed a range of estimated values differing by up to 27%. The predicted number-average molecular weight is compared to the corresponding experimental measurements as well as their weight-average counterparts in Figure 3. The model reproduces the measured number-average molecular weights with good accuracy. Calculations have also been carried out for non-optimal experimental datasets, leading to equally good agreement between measurements and model predictions (with different, but generally similar optimal values of the rate and water removal parameters). Both experimental data and modeling predictions exhibit an almost steady increase in molecular weight with time following a relatively protracted period of slow growth—a rather counterintuitive finding, as one would expect chain growth to gradually slow down and degree of polymerization to converge to a limiting value.

It can be argued that the actual trend, concealed by experimental errors and premature interruption of the process, should look more like a sigmoid function converging to a very high yet finite molecular weight. This scenario sounds more convincing when looking at the weight-average experimental values, which cannot be predicted by the simplified version of our model. Numerical predictions of number-average molecular weight, on the other hand, tend to grow linearly with time, as verified by running the simulation for longer time scales. This is indeed what happens when water concentration falls to negligible levels as we can easily verify by setting *w* = 0. Then, the set of rate equations is simplified to $\frac{dc}{dt}=k_{1}a^{2}$ and $\frac{da}{dt}=-k_{1}a^{2}$, which is trivial to solve analytically yielding the solution $a\left(\tau \right)=\frac{a_{w\to 0}}{1+k_{1}a_{w\to 0}\tau }$ and $c\left(\tau \right)=\frac{k_{1}a_{w\to 0}^{2}\tau }{1+k_{1}\tau }$, and a predicted number-average molecular weight $MW\left(\tau \right)=\frac{c\left(\tau \right)}{a\left(\tau \right)}{\mathrm{FW}}_{\mathrm{mono}}={\mathrm{FW}}_{\mathrm{mono}}k_{1}a_{w\to 0}\tau$, where we have used the symbols $a_{w\to 0}$ and ${\tau =t-t}_{w\to 0}$ to emphasise that the above approximation is valid at a time $t_{w\to 0}$ when water concentration has become negligible and the initial condition for *a* becomes ${a(t_{w\to 0})=a}_{w\to 0}\ll a_{0}$.

In other words, chain growth in the absence of water is predicted to obey a linear trend and proceed uninterrupted until reaching the physical limit of depleting all available pairs of -OH and -COOH groups in the mixture (this highly improbable and idealized termination mechanism could involve the formation of one or more very long ring polymers neutralizing all remaining reactive groups). 

In practice, the prevalent presence of very long chains in the mixture would bring to the fore mechanisms related to mass transport (low diffusivity) of the emerging polymer species, affecting the overall kinetics in manners that were not considered when setting up our model. The increasing impact of these mechanisms on the mixture’s kinetics could indeed lead to sublinear dependence of molecular weight on time. The number of our experimental data is too small to allow more complex modeling without overfitting. However, reasonable assumptions and simplifications can be used to illustrate the impact of diffusion-controlled mechanisms. Mass-transport effects are expected to arise when chain size exceeds the entanglement molecular weight of PLLA, which is in the range of 9000 g/mol [58]. Assuming constant *k*_−1_, the polymerization rate constant would be expected to exhibit a sigmoid dependence on molecular weight. In a simplified approach, we adopt a stepwise change; as an example, we try a 10-fold decrease when *M*_n_ exceeds the entanglement molecular weight. An optimization calculation under these assumptions yielded similar values for *k*_−1_ and *k*_w_ as in Table 1, but *k*_−1_ was about 10 times lower. The objective function, *F*, is more sensitive to errors in the range of high molecular weights, and the depolymerization constant ‘adapted’ to the decrease in the *k*_1_ to keep the total error low. The linear trend with time was preserved but a sublinear one cannot be ruled out if more complex diffusion-related mechanisms are present. Regardless of the above scenaria that concern time scales even longer than our experiments, it is useful to look at the change of water content with time as a means of reevaluating the applicability of our assumptions about the constancy of the kinetic parameter governing water removal. Whether the scenario of negligible water concentration holds can be examined by looking at the concentrations calculated as functions of time. 

**Figure 4:**
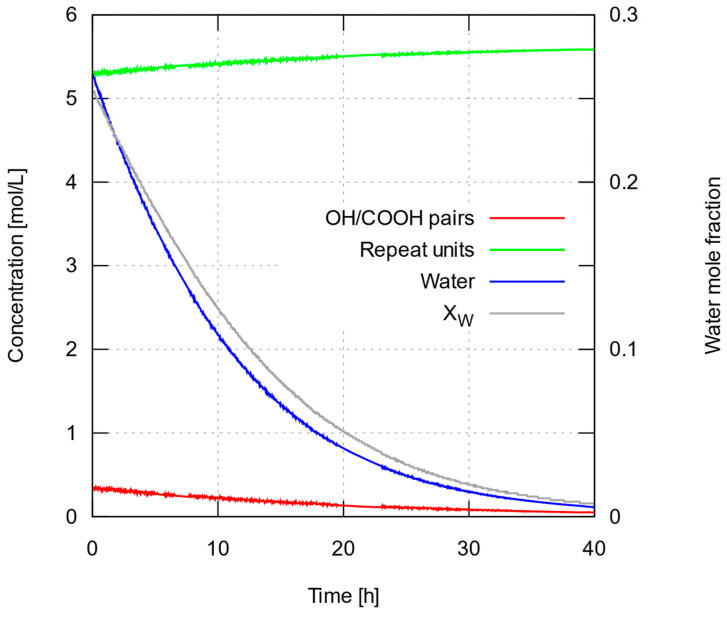
Calculated concentrations *a*, *c*, *w* (see main text) and water mole fraction *x*_w_ with time, when using rate and water removal constants that have been optimized for predicted number-average molecular weight to match experimental data. Results shown for the upper bound of estimated initial dry LA mass, 7.11 g (very similar results were obtained for the other two estimated values of LA mass; see main text for details).

Concentrations of P-A (and Q-B) bonds, repeat units (equal in number to the ester bonds formed during polymerization) and water, as well as water’s mole fraction with time, are shown in Figure 4. It can be readily seen that the calculated water content is far from negligible during the slow-growth period (roughly, the first 40 h) but remains at very low levels afterwards resulting in the predicted quasi-linear trend, which appears to match the experimental data quite well. On the other hand, the wide range of change in water content breaks the assumption of small, relatively stable water concentration made to justify a constant value for the water removal coefficient, *k*_w_. Then, we expect the model to behave during the slow-growth period in one of the following ways: (i) water content in actual experiments remaining high for a prolonged period: in this case, the chemical equilibrium would shift towards depolymerization and our model would overestimate the molecular weight throughout the run; (ii) water content falling rapidly to very low levels: should this be the case, the equilibrium would shift towards polymerization early on, and our model would underestimate the molecular weight throughout the simulation; (iii) water content falling at a rate comparable to the one predicted numerically: in this last case, the model is expected to work well at long enough time scales but would tend to deviate more strongly (in terms of fractional errors) during the slow-growth period. This is indeed the case, as numerical predictions in Figure 3 vary more smoothly than the measured data, and tend to overestimate molecular weight for up to about the first 50 h. A more sophisticated model would account for a water removal coefficient, *k*_w_, varying with water content, allowing a scenario in which water content would decrease slowly until falling under some ‘critical’ value, thus shifting equilibrium towards polymerization and accelerating chain growth. 

Another interesting observation has to do with the rapid change of concentrations during the first few steps of the simulation. Keeping in mind that the concentrations refer to bonds that take part in the polymerization–depolymerization reaction, it is only at the beginning of the simulation that the concentration variable *a* coincides with the initial lactic acid concentration. The curves in Figure 4 suggest that a large fraction of the lactic acid molecules (more than 90%) react immediately to form dimers and higher oligomers. The average degree of polymerization during this very early stage is about 15 (estimated as *c*/*a*, not quite far from the ten-mers measured by GPC during the first 20 h of experiment under optimal conditions). The concentration of available pairs of reactive -OH and -COOH groups providing P-A and Q-B bonds falls by that same ratio, and the corresponding amount of water is produced; these two factors immediately restrict chain growth until enough water is removed and the equilibrium is shifted towards polymerization.

Finally, it is worth discussing the role of the equilibrium constant *k*_eq_ = *k*_1_/*k*_−1_ as compared to the impact of the magnitude of the rate constants themselves (Table 1). We multiplied the rate constants, *k*_1_ and *k*_−1_, either by the same factor or by different ones, and calculated three measures of our predictions conforming to the experimental data, i.e., the sum of square errors (our objective function, *F*), the sum of fractional errors squared (as percentages of the experimental values) and the sum of absolute fractional differences. It was found that when decreasing the rate constants in a uniform manner even by orders of magnitude (multiplying by down to 10^−3^), the objective function remained largely unaffected (increased negligibly) whereas the other two measures improved (decreased) further, albeit by a very small amount. In other words, the values of the rate constants themselves play a minor role in reproducing the polymerization data as long as they have the same ratio, i.e., equilibrium constant—which is not a surprising result.

It should be noted that the fractional measures could improve even further by decreasing the polymerization rate constant and/or increasing the depolymerization rate constant; however, the objective function would increase greatly as the model would fail to capture the long-term dynamics of the system (high molecular weights). For instance, increasing *k*_1_ with *k*_−1_ fixed, would yield overestimated long-term predictions of number-average molecular weight. A uniform change of the rate constants, on the other hand, implies that the existing balance is preserved between chain formation and chain destruction. For instance, doubling both rate constants results in twice faster chain building, but also twice faster chain destruction. In view of the above, the optimal values of the rate constants should be understood as upper bounds of a range of values of *k*_1_ and *k*_−1_, constrained by the condition of an optimal equilibrium constant, *k*_eq_ = *k*_1_/*k*_−1_; on top of them, comes the optimal value (Table 1) for the water removal parameter, *k*_w_, that fills the set of estimated values for the parameters of our model.

## 4. Conclusions

We have implemented a process for the synthesis of poly(L-lactic acid) via catalytic polycondensation carried out under relatively mild conditions, with the aid of azeotropic dehydration to shift the chemical equilibrium towards polymer formation. To optimize this process, lactic acid and catalyst (tin octoate) concentration in dry toluene, as well as the temperature, were systematically varied. Under the optimal conditions, i.e., lactic acid concentration 58.4 wt.% with respect to toluene and catalyst concentration 0.56 wt.% with respect to the lactic acid, the polymerization was found to proceed at temperatures between 134 and 137 °C and prolonged reaction times (ca. 120 h). Under these conditions, PLLAs with weight-average molecular weight of as high as 161 kDa and dispersity 1.81 were isolated at near quantitative yields.

We have also developed a simplified kinetic model that can capture the observed experimental kinetics when adjusting for the rate constants and an effective water removal rate parameter. Our approach relied on the same assumptions as a previous model by Harshe et al. [40], which was capable of predicting the whole molecular weight distribution with time, albeit at a price in terms of required computational resources. A reinterpretation of polycondensation as a reaction among bonds that exchange their constituent groups allowed the recasting of the model in a considerably simplified form of just three differential equations, which does not require any arbitrary upper bound to the degree of polymerization and can calculate the number-average molecular weight with time at lightning speed (at the cost of giving up predictions of molecular weight distribution and weight-average values). This fast numerical scheme allowed us to determine optimal values of the three molecular-weight-independent rate constants entering the equations, and obtain results that match the experimental measurements of number-average molecular weight with quite satisfactory accuracy. Notably, it would be particularly easy to extend the model for the study of the process across wider temperature ranges by adding an Arrhenius-type temperature dependence to the rate constants (certain assumptions will have to be made as regards the water-removal effective rate constant). The problem size will remain feasible provided enough data points are given for each temperature and optimal values for the activation energies would be easy to determine. 

The PLLA polycondensation setup presented in this work is aimed at the production of PLLA from recycled food waste that has been converted to lactic acid through an existing fermentation process. The conditions achieved during the process satisfy local regulations and allow reduced operating costs. Furthermore, our kinetic model can assist in the design of the process during the subsequent scale-up steps by simplifying the calculations and allowing quick comparison of alternative scenaria based on data from pilot-plant production runs. The way is thus paved to the development of environmentally friendly and economically feasible processes in terms of operating conditions, valorization of recycled waste and production of recyclable bioplastic materials. 

## Figures and Tables

**Table 1:** Optimal rate constants.

Polymerization Rate Constant, *k*_1_ [L mol^−1^ s^−1^] ^[a]^	Depolymerization Rate Constant, *k*_−1_ [L mol^−1^ s^−1^] ^[a]^	Equilibrium Constant *k*_eq_ = *k*_1_/*k*_−1_	Water Removal Effective Coefficient, *k*_w_
For lower bound of estimated initial dry LA mass (5.58 g)			
≤15.94	≤6.48 × 10^−2^	246.0	4.34 × 10^−4^
For intermediate estimated initial dry LA mass (6.75 g)			
≤16.47	≤7.14 × 10^−2^	230.7	4.76 × 10^−4^
For upper bound of estimated initial dry LA mass (7.11 g)			
≤15.06	<6.47 × 10^−2^	232.8	4.85 × 10^−4^

^[a]^ In each case of estimated initial dry LA mass, the table entries for the polymerization and depolymerization rate constants are upper bounds to the values of these parameters, under the condition that they give the indicated equilibrium value. Units of mole refer to bonds (see main text for details).

## Data Availability

Data will be available upon request to the corresponding author.

## Supplementary Materials

The following supporting information can be downloaded at: https://www.mdpi.com/article/10.3390/polym15234569/s1, Figure S1: ^1^H-NMR of the reaction mixture after the first dehydration step (a). The 5% *w*/*w* of lactic acid were converted to L-lactide; Figure S2: ^1^H-NMR of the PLA formed by the polycondensation of L-lactic acid; Figure S3: ^13^C-NMR of the PLA formed by the polycondensation of L-lactic acid; Figure S4: ^1^H-NMR of the PLA formed by the polycondensation of DL-lactic acid; Figure S5: ^13^C-NMR of the PLA formed by the polycondensation of DL-lactic acid; Figure S6: Kinetic studies for the direct polycondensation of DL-lactic acid; Figure S7: Investigation of the PLA chain extension using 1,4-phenylene diisocyanate (PDI) as the cross-linker; Figure S8: Monitoring the growth of PLA by GPC, following the optimum polymerization conditions; Figure S9: Infrared spectra of PLA; Figure S10: TGA thermograms (left), dTG curves (middle) at a heating rate of 20 °C/min under a nitrogen atmosphere; Data analysis (right) of 5% weight loss (T_0.05_), 50% weight loss (T_0.50_) and maximum temperature decomposition (Td, max). PLA at different reaction conditions; Figure S11: Differential scanning calorimetry (DSC) graphs of PLA; Table S1. Initial conditions used in calculations to determine rate constants, and modeling predictions; example using the upper bound of estimated initial LA dry mass; Table S2. Optimization of the polycondensation reaction; Table S3. IR absorption peaks of PLA synthesized by direct polycondensation of LA; Table S4. Differential scanning calorimetry (DSC) of PLA. References [24,32,48,50] are cited in Supplementary materials.

## Appendix A. Summary of Notation

**Symbol**	**Meaning**	**Symbol**	**Meaning**
*a*	Molar concentration of HOOCR_i_-OH bonds connecting an end hydroxyl group with the rest of a PLA chain	*M*_n_	Number-average molecular weight
*b*	Molar concentration of HOR_j_COO-H bonds connecting the hydrogen atom of an end carboxyl group with the rest of a PLA chain	*M*_w_	Weight-average molecular weight
*c*	Molar concentration of bonds of a HOOCR_i_- subchain to a HOR_j_COO- one, coming about when a HOOCR_i_-OH bond and a HOR_j_COO-H one exchange their constituent groups.	*N*	Number of measurements of molecular weight during PLA polycondensation experiment
*C*	Molar concentration of catalyst in reactant mixture	*P_i_*	Molar concentration of polymer chains with degree of polymerization equal to i.
*F*	Objective function defined as the sum of squares of the deviations of modeling predictions from experimental measurements of number-average molecular weight.	*w*	Molar concentration of H-OH bonds that link a hydrogen atom with a hydroxyl group to form water, and come about when a HOOCR_i_-OH bond and a HOR_j_COO-H one exchange their constituent groups.
*h*	Step used by the numerical solver of the kinetic differential equations	*W*	Molar concentration of water
*k*_1_	Polymerization rate constant	*S*	Molar concentration of solvent in reactant mixture
*k*_−1_	Depolymerization rate constant	*x*_w_	Water mole fraction
*k*_w_	Effective rate constant associated with water removal	*t*	Time (of experimental measurements and simulation steps, alike)
