# Supplementary material for: Synthesis and Modeling of Poly(L-lactic acid) via Polycondensation of L-Lactic Acid
Source: Polymers (Basel). 2023 Nov 29;15(23):4569. doi: 10.3390/polym15234569 (PMC10708065; doi:10.3390/polym15234569)
Supplement: Supplementary file 1 [file polymers-15-04569-s001.zip › polymers-2640863-supplementary.pdf]

Synthesis and Modeling of Poly(L-lactic acid) via Polycondensation of L-Lactic Acid

Alexis Theodorou [1], Vasilios Raptis [2,3], Chrissie Isabella Maria Baltzaki [1], Thrassyvoulos Manios [4], Vagelis Harmandaris [2,5] and Kelly Velonia[1]

Supplementary Material

1. Modeling

Table S1. Initial conditions used in calculations to determine rate constants, and modeling predictions; example using the upper bound of estimated initial LA dry mass (see main text for details).

Experimental Data

LA dry mass in g	**= 7.11 (upper bound)**
Toluene as solvent, mass in g	= 10.44
Toluene for catalyst, volume in ml	= 2.0
Catalyst mass in g	= 0.04

Other Data

LA MW, in g/mol	= 90.08
Toluene MW, in g/mol	= 92.14
Tin octoate (catalyst) MW, in g/mol	= 405.12
LA density in g/ml	= 1.21
Toluene density in g/ml	= 0.87

Assumptions (approximation of total volume)

Total Volume of Solution, in litres, V =	[(Toluene-for-catalyst volume, in ml) + (Toluene-as-solvent, volume in ml)] / 1000

Negligible initial concentration of water

Calculation of initial concentrations

Toluene-as-solvent volume =	(Toluene as solvent) / (Toluene density) =	10.44 / 0.87 =	12 (mL)
Total Volume of Solution, V =	(12 + 2) / 1000 =	(by approximation)	0.014 (L)
Toluene for catalyst (mass, in g)	(Toluene for catalyst, volume in ml) * (Toluene density) =	2.0 * 0.87 =	1.74 g
Toluene moles =	((Toluene for catalyst, mass) + (Toluene as solvent, mass)) / (Toluene Mol Weight) =	(1.74 + 10.44) / 92.14 =	0.13219

**[Toluene]** =	(Toluene moles) / V =	0.13219 / 0.014 =	**9.4422 (M)**
LA moles =	(LA dry mass) / (LA MW) =	7.11 / 90.08 =	0.0789*2984*
**[LA]** =	(LA moles) / V (total volume of toluene, L) =	0.0789 / 0.014 =	**5.6378 (M)**
Catalyst moles =	(Catalyst mass) / (Tin Octoate MW) =	0.04/405.12 =	0.0000198736
**[Catalyst]** =	(Catalyst moles) / V (total volume of toluene, L) =	0.0000198736/ 0.014	**0.0007053 (M)**

2. Materials

Chemicals were purchased from Fluka (DL-lactic acid), Fisher Chemical (L-lactic acid), Sigma-Aldrich (tin octoate (Tin(II) 2-ethylhexanoate), toluene and Molecular Sieves 3 Å) and used as received unless otherwise noted.

3. Analytical Techniques

Gel Permeation Chromatography (GPC)

Gel Permeation Chromatography (GPC) was performed on a Shimadzu modular system comprising a CBM-20A system controller, a LC-20AD pump (flow rate at 1 $mL \cdot min^{-1}$), a SIL-20A automatic injector, a 10.0 μm bead-size guard column (50 × 7.5 mm) followed by PLgel Mixed-D column (300 × 7.5 mm, bead size: 5 μm, pore size maximum: 5000 Å), a SPD-20A ultraviolet detector, and a RID-20A differential refractive index detector. The temperature of the column was maintained at 40 °C using a CTO20AC oven. HPLC grade THF was used as eluent. Calibration was performed using commercial narrow molecular weight distribution polystyrene standards with molecular weights ranging from 580 to 299400 $g \cdot mol^{-1}$ (Polymer Laboratories). Before injection, all samples were passed through 0.45 μm filters. All reported molecular weights were calculated by comparing with polystyrene standards and therefore the MW provided relate to polystyrene of the same hydrodynamic volume.

NMR Spectroscopy

^{1}H and ^{13}C NMR spectra were recorded on a Bruker 500 MHz Avance spectrometer. All chemical shifts are reported in ppm (δ) relative to tetramethylsilane, referenced to the chemical shifts of residual $CHCl_3$-d resonances (^{1}H and ^{13}C). The following abbreviations were used to explain the multiplicities: s = singlet, bs = broad singlet, d = doublet, t = triplet, m = multiplet.

IR Spectroscopy

Infrared spectroscopy was performed with a Nicolet 6700 Attenuated Total Reflection Fourier Transform Infrared (ATR FT-IR) spectrometer using Omnic (Thermo Electron Corporation) software.

Thermogravimetric analysis (TGA)

The thermogravimetric analyses were performed using Perkin Elmer Pyris Diamond TG/DTA Instrument. In a typical measurement dried samples (initial weight ≤ 5mg) set in alumina pans, were placed on a thermobalance, and heated between 25 and 550 °C at a heating rate of 10 °C/min under continuous nitrogen atmosphere.

Differential Scanning Calorimetry (DSC)

Differential scanning calorimetry (DSC) was carried out using a PerkinElmer DSC. The DSC measurements were performed in a nitrogen atmosphere from 20 to 200 °C at a heating rate of 20 °C /min, melted at 200 °C and kept 2 min, then cooled to 40 °C at a cooling rate of 20 °C/min. The samples were analyzed in two heating scans.

4. Experimental section

4.1 General procedure for the synthesis of PLA through direct polycondensation of Lactic Acid[1]

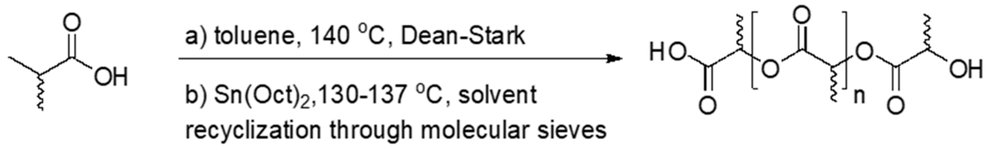

Commercial D,L- or L-lactic acid (8.08 g, 78.9 mmol, 12% water residual) and a stirring bar were placed in a two-neck 100 mL round bottom flask equipped with a Dean-Stark receiver with 15 ml capacity and a condenser. Toluene (25 mL), predried with molecular sieves, was added and the resulting biphasic mixture was heated at 140 °C for 2 hours (Step **A**). At the end of Step A, the temperature was set at 130 °C and 1.2 mL distilled water and 13 mL toluene were removed via the Dean Stark apparatus. The Dean Stark was then replaced by a glass tube containing 8 g of activated molecular sieves (3 Å) and the system was purged with nitrogen. During refluxing toluene, 2 ml of a 20 mg/mL solution of tin octanoate (0.025 mmol) in dry toluene were added to initiate the polymerization. Azeotropic dehydration was carried out for 48-120 hours at 130-137 °C (Step **B**). After cooling, the resulting viscous mixture was dissolved in 15 mL chloroform by means of heat and/or sonication. PLA was precipitated with cold MeOH (100 mL). The precipitate was filtered, washed with 15 mL cold MeOH and finally dried *in vacuo*. PLA was recovered as a white powder of PLA in 80-86% yield and characterized with NMR, GPC, IR, TGA and DSC.

The polycondensation reaction was optimized by means of varying LA and catalyst concentration as well as the temperature as described in Table S2, below.

Table S2. Optimization of the polycondensation reaction.

Entry	*L*-LA wt. % [a]	$Sn(Oct)_2$ wt. % [b]	Toluene (mL)	Reaction Time (h)	Temperature (°C)	M_w	M_n	Đ M_w/M_n
1	58.4	0.56	14	92	134-137	78640	41186	1.91
2	58.4	0.56	14	120	134-137	149452	81146	1.84
3	91.1	0.56	9	120	134-137	117248	59015	2.00
4	43.1	0.56	19	120	134-137	106127	55906	1.89
5	22.8	0.79	36	120	134-137	109741	43236	2.54
6	22.8	0.79	36	120	134-137	166220	96435	1.72[c]
7	22.8	0.79	36	120	134-137	94717	70323	1.35[d]
8	58.4	0.56	14	120	130-133	83869	38113	2.20
9[e], [f]	12.4	0.40	66	116	130-133	1607	1190	1.55
10[f]	12.4	0.80	66	120	130-133	8113	5764	1.41

[a] All concentrations refer to the second step; L-Lactic acid contains 10-12% residual water, wt. %: g of L-lactic acid per 100 g toluene; [b] wt. %: g of $Sn(Oct)_2$ per 100 g of L-Lactic acid; The PLA chain extension was investigated in the presence of the crosslinker 1,4-phenylene diisocyanate (PDI). 30 mg of PDI (0.4 wt. %) were added and the reaction mixture was further heated for [c] 20 min and [d] 48 hours; [e] The reaction was performed without solvent recyclization [f] D,L-lactic acid.

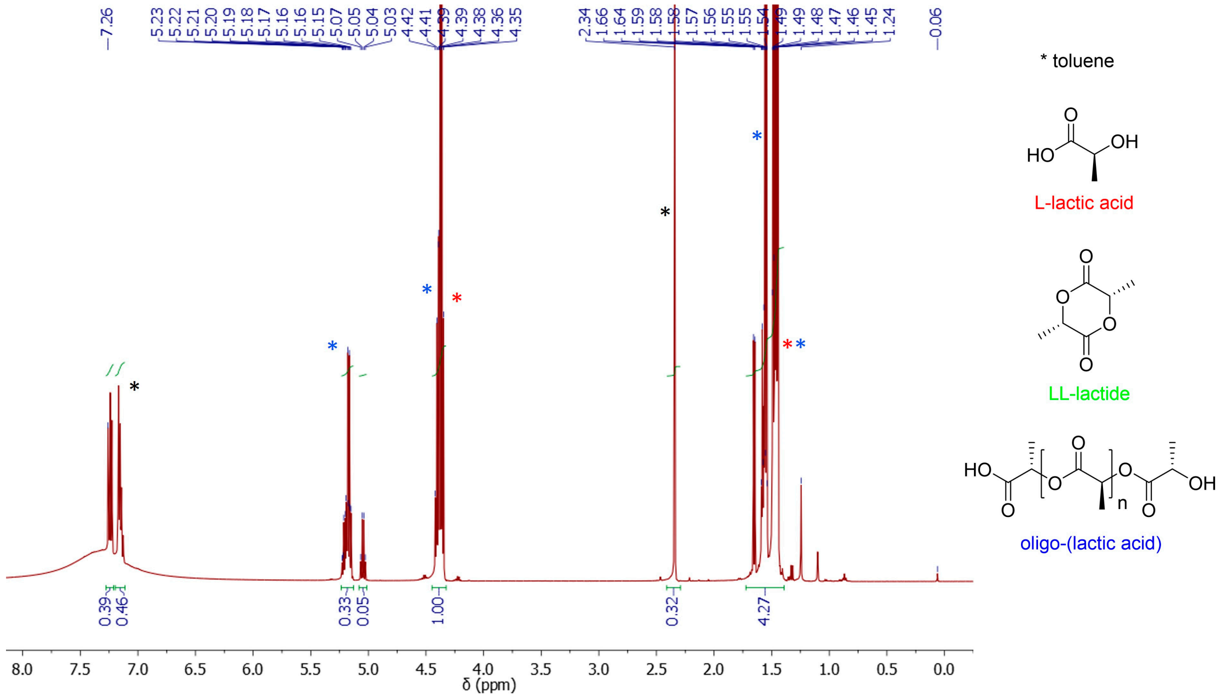

Figure S1. ^{1}H-NMR of the reaction mixture after the first dehydration step (**a**). The 5% w/w of lactic acid were converted to L-lactide. ^{1}H NMR (500 MHz, $CDCl_3$): δ 5.23-5.13 (m, 0.33H, COCH, oligo(LA)), 5.05 (q, J = 6.7 Hz, 0.05H, COCH, LL-lactide), 4.44-4.33 (m, 1H, HOCH, LA and oligo(LA)), 1.66-1.43 (m, 4.15H, CH_3, LL-lactide, LA and oligo(LA).

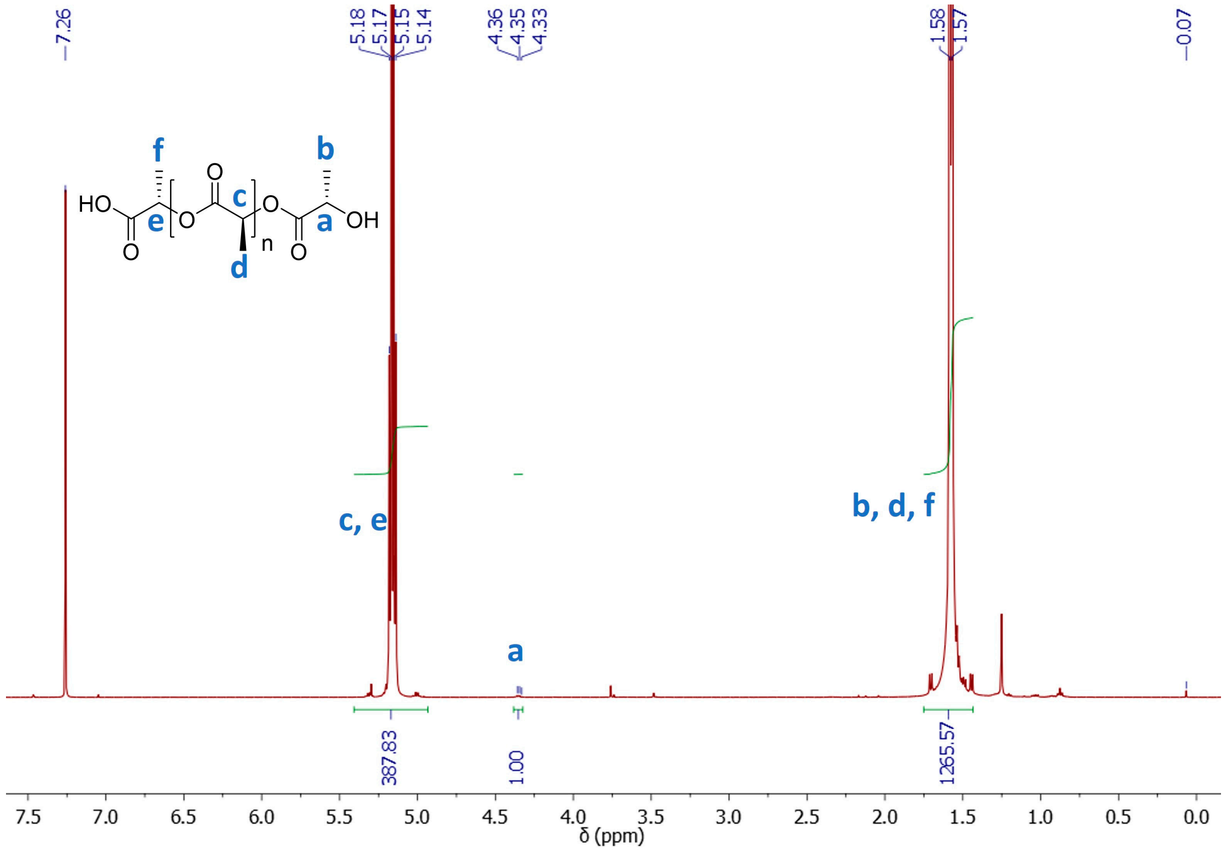

Figure S2. ^{1}H-NMR of the PLA formed by the polycondensation of L-lactic acid. Reaction conditions: L-lactic 58.4 wt. %, 0.56 wt. % catalyst at 134-137 °C (Table S2, Entry 2). ^{1}H NMR (500 MHz, $CDCl_3$): δ 5.10 (q, *J* = 7.1 Hz, 387H, COCH), 4.29 (dd, *J* = 13.8 and 6.9 Hz, 1H, HOC*H*), 1.52 (d, *J* = 7.1 Hz, 1161H, CH_3).

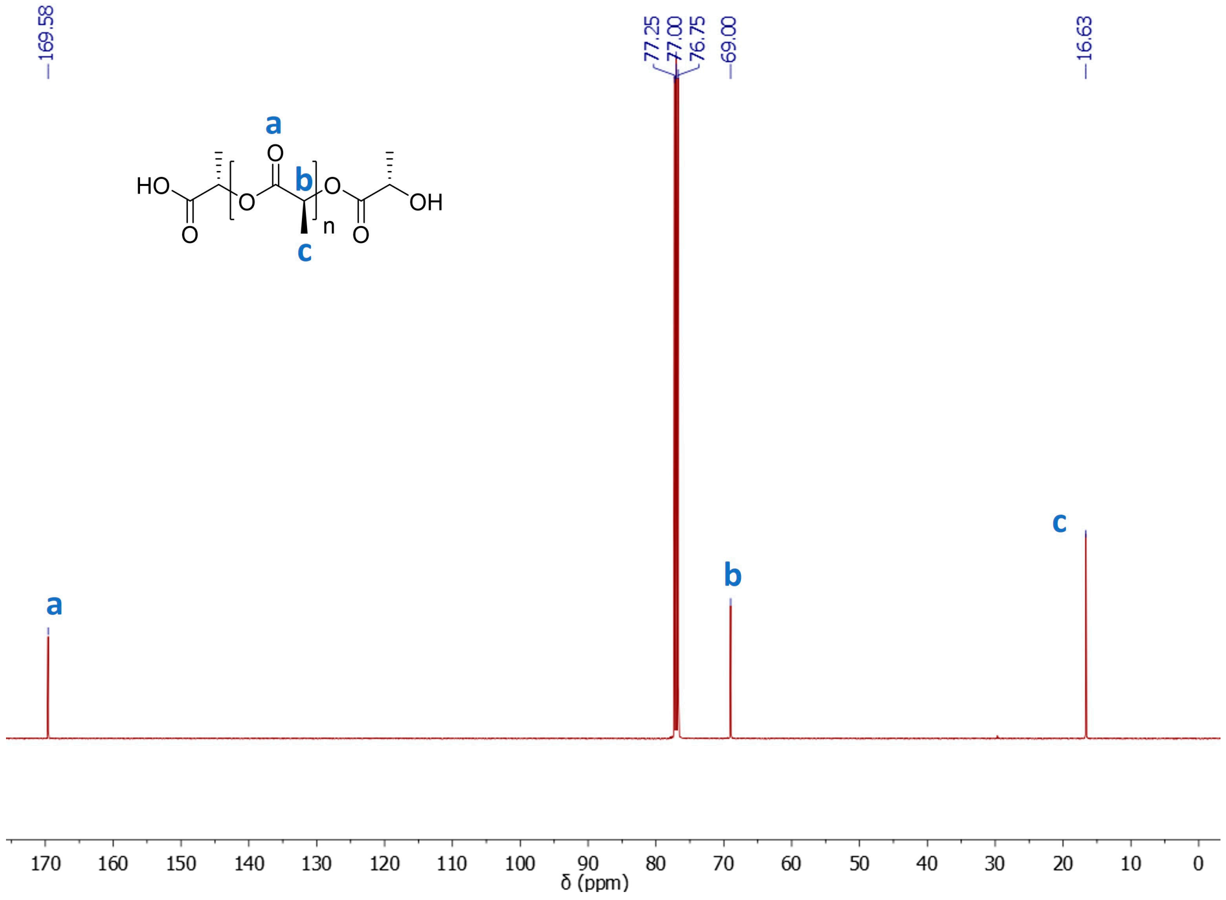

Figure S3. ^{13}C-NMR of the PLA formed by the polycondensation of L-lactic acid. Reaction conditions: L-lactic 58.4 wt. %, 0.56 wt. % catalyst at 134-137 °C (Table S2, Entry 2). ^{13}C NMR (126 MHz, $CDCl_3$): δ 169.6 (C=O), 69.0 (CH), 16.6 (CH_3).

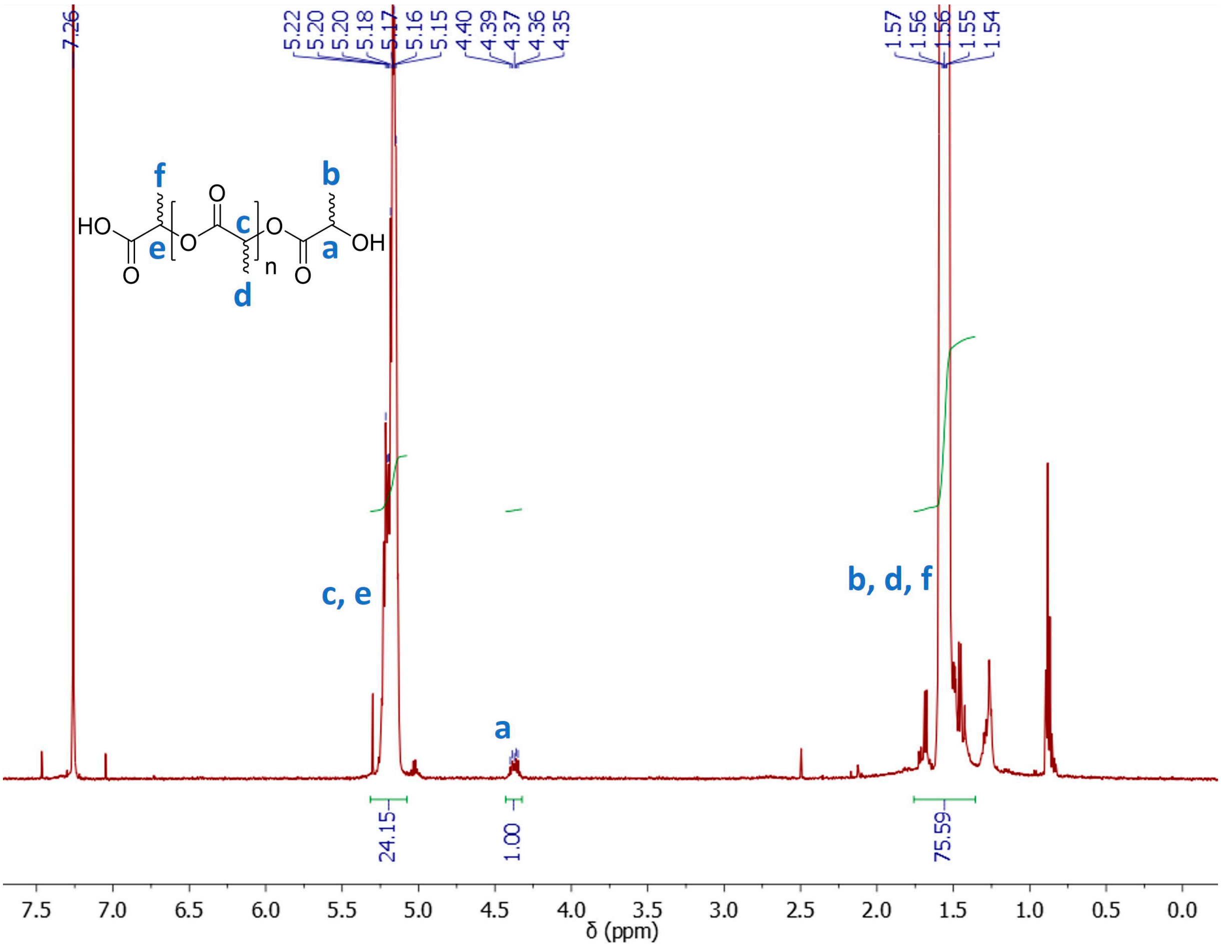

Figure S4. ^{1}H-NMR of the PLA formed by the polycondensation of DL-lactic acid. Reaction conditions: DL-lactic 12.4 wt. %, 0.8 wt. % catalyst at 130-133 °C (Table S2, Entry 10). ^{1}H NMR (500 MHz, $CDCl_3$): δ 5.30-5.13 (m, 24H, COCH), 4.42-4.32 (m, 1H, HOC*H*), 1.72-1.43 (m, 75H, CH_3).

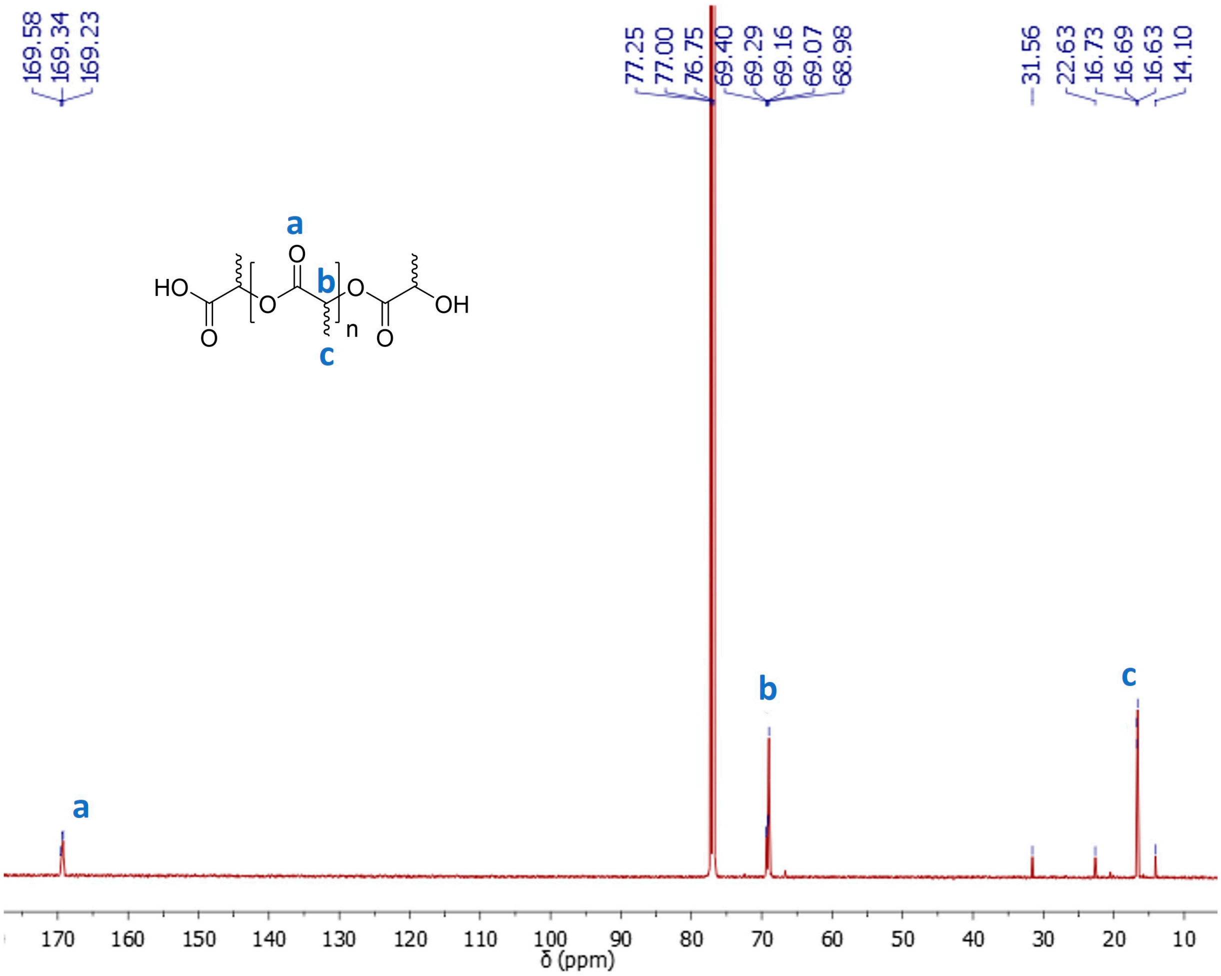

Figure S5. ^{13}C-NMR of the PLA formed by the polycondensation of DL-lactic acid. Reaction conditions: DL-lactic 12.4 wt. %, 0.8 wt. % catalyst at 130-133 °C (Table S2, Entry 10). ^{13}C NMR (126 MHz, $CDCl_3$): δ 169.6, 169.3, 169.2 (C=O), 69.4, 69.3, 69.2, 69.1, 69.0 (CH), 16.6 (CH_3).

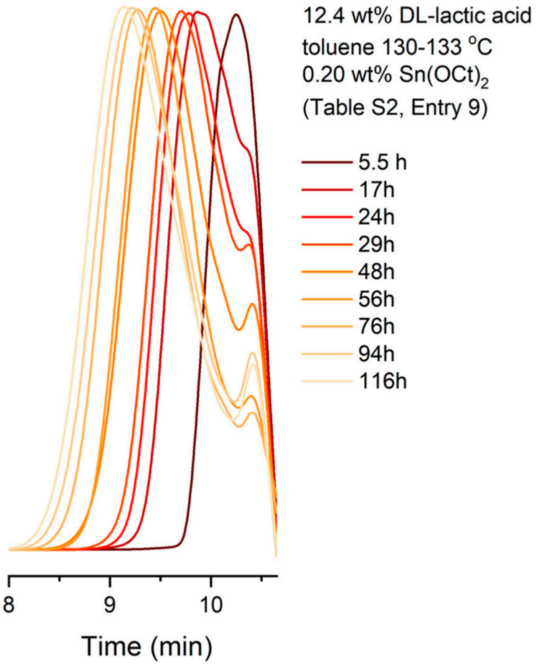

Time (hours)	Number Average Molecular Weight (M_n)	Weight Average Molecular Weight (M_w)	Polydispersity Đ M_w/M_n
5.5	295	317	1.07 [a]
17	374	461	1.23[a]
24	418	546	1.30[a]
29	436	585	1.34[a]
48	524	830	1.43
56	636	916	1.58
76	724	1163	1.60
94	726	1256	1.73
116	1190	1607	1.55

Figure S6. Kinetic studies for the direct polycondensation of DL-lactic acid. Reaction conditions: DL-lactic 12.4 wt. %, 0.4 wt. % catalyst at 130-133 °C, without solvent dehydration (Table S2, Entry 9). GPC graphs (*left*) and data analysis (*right*) of aliquots (0.5 mL) withdrawn by the reaction mixture during the polymerization. [a] Flagged as suspect data as the measured M_n is beyond the calibration curve in GPC.

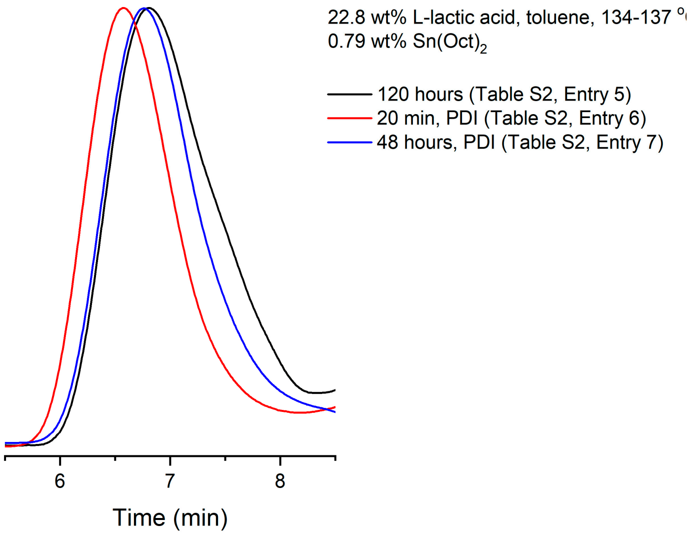

Figure S7. Investigation of the PLA chain extension using 1,4-phenylene diisocyanate (PDI) as the crosslinker. GPC graphs; black trace: 120 min reaction time, no PDI (43 kDa, Table S2, Entry 5), red trace: 20 min after the addition of PDI (96 kDa, Table S2, Entry 6), blue trace: 48 hours after the addition of PDI (70 kDa, Table S2, Entry 7).

3.2 Polycondensation of L-lactic acid following the optimum reaction conditions (Table S2, Entry 2)

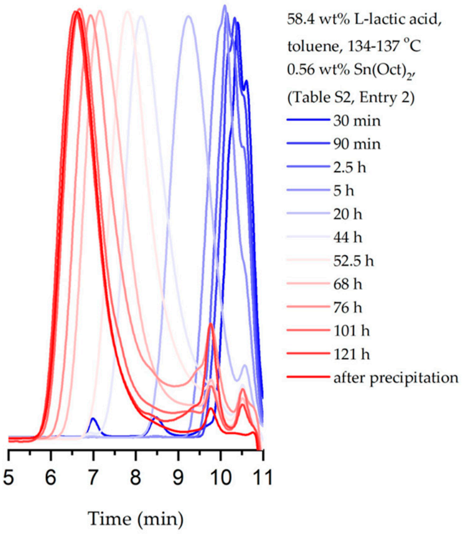

Reaction Time (hours)	Number Average Molecular Weight (M_n)	Weight Average Molecular Weight (M_w)	Polydispersity Đ M_w/M_n
0.5	217	243	1.12 [a]
1.5	246	283	1.15[a]
2.5	272	318	1.17[a]
5	341	422	1.24[a]
20	947	1440	1.52
44	4141	8198	1.99
52.5	5766	13866	2.40
68	22912	49359	2.15
76	36306	78045	2.15
101	58856	130141	2.21
121	83817	161127	1.92
121[b]	81146	149452	1.84

Figure S8. Monitoring the growth of PLA by GPC, following the optimum polymerization conditions: L-lactic acid 58.4 wt. %, 0.56 wt. % catalyst at 134-137 °C (Table S2, Entry 2). GPC graphs (left) and data analysis (right) of aliquots (0.5 mL) withdrawn from the reaction mixture at specific time points during polymerization and, after isolation. [a] Suspicious-flagged data as the measured M_n is beyond the calibration curve in GPC.

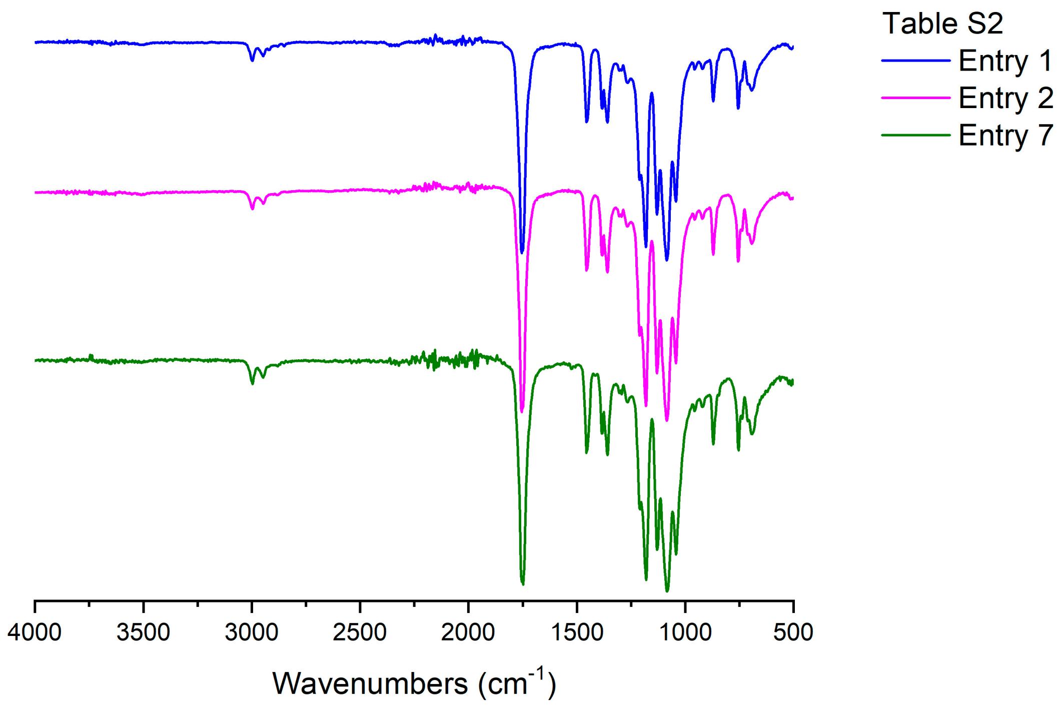

Figure S9. Infrared spectra of PLA. L-lactic 58.4 wt. %, 0.56 wt. % catalyst at 134-137 °C at 92 hours (Table S2, Entry 1) and 120 hours (Table S2, Entry 2). L-lactic 22.8 wt. %, 0.79 wt. % catalyst at 134-137 °C heated for 120 hours and followed by the addition of 0.4 wt. % PDI. The reaction was left stirring at the same temperature for 48 hours (Table S2, Entry 7).

Table S3. IR absorption peaks of PLA synthesized by direct polycondensation of LA[2-5]

Entry (Table S1)	-CH- stretch	-C=O carbonyl stretch	-NH- stretch	$-CH_3$ bend	-CH- deformation	-C=O bend	-C-O stretch	-OH bend	-C-C- stretch	Amorphous phase	Crystalline phase
1	2999.5<br>2943.8	1754.4	-	1454.5	1384.3<br>1359.2	1266.7	1210.7<br>1182.7<br>1129.1<br>1084.5	1042.3	955.5<br>921.8	871.5	756.6
2	2996.2<br>2947.5	1756.3	-	1456.0	1381.5<br>1359	1267.6	1210.7<br>1179.8<br>1129.1<br>1084.5	1042.3	955.5<br>918.9	871.5	753.8
7	2993.2<br>2946.6	1754.9	1525.7	1451.8	1383.1<br>1358.6	1265.2	1209.9<br>1180.9<br>1128.3<br>1083.3	1041.3	959.7<br>922.8	870.1	754.2

3.3 Thermogravimetric analysis (TGA), differential scanning calorimetry (DSC) and infrared spectroscopy (IR) of PLA (Table S2, Entries, 1, 2 and 7)

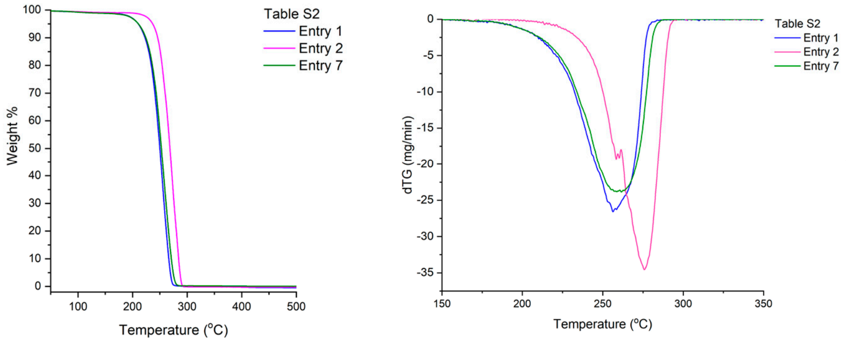

Entry (Table S2)	$T_{0.05}$ (°C)	$T_{0.50}$ (°C)	$T_{d, max}$ (°C)
1	210.4	249.9	278.1
2	236.9	268.4	296.5
7	210.8	252.8	283.7

Figure S10. TGA thermograms (*left*), dTG curves (*middle*) at a heating rate of 20 °C /min under a nitrogen atmosphere; Data analysis (*right*) of 5% weight loss ($T_{0.05}$), 50% weight loss ($T_{0.50}$) and maximum temperature decomposition ($T_{d, max}$). PLA at different reaction conditions: L-lactic 58.4 wt. %, 0.56 wt. % catalyst at 134-137 °C at 92 hours (Table S2, Entry 1) and 120 hours (Table S2, Entry 2). L-lactic 22.8 wt. %, 0.79 wt. % catalyst at 134-137 °C heated for 120 hours and followed by the addition of 0.4 wt. % PDI. The reaction was left stirring at the same temperature for 48 hours (Table S2, Entry 7).

Table S4. Differential scanning calorimetry (DSC) of PLA.

Entry (Table S1)	T_{cc}	T_{m1} (°C)	T_{m2} (°C)	T_g (°C)	T_c	ΔH_m[a]	Crystallinity (%)
1	106.4	162.5	170.2	59.9	-	16.9	18.0
2	129.1	171.6	-	65.2	-	15.8	16.9
7	106.4	158.6	165.9	61.1	-	27.5	29.3

[a] The standard melting enthalpy (ΔH°_m) of a 100% crystalline PLA is equal to 93.7 J × g^{-1}.[6]

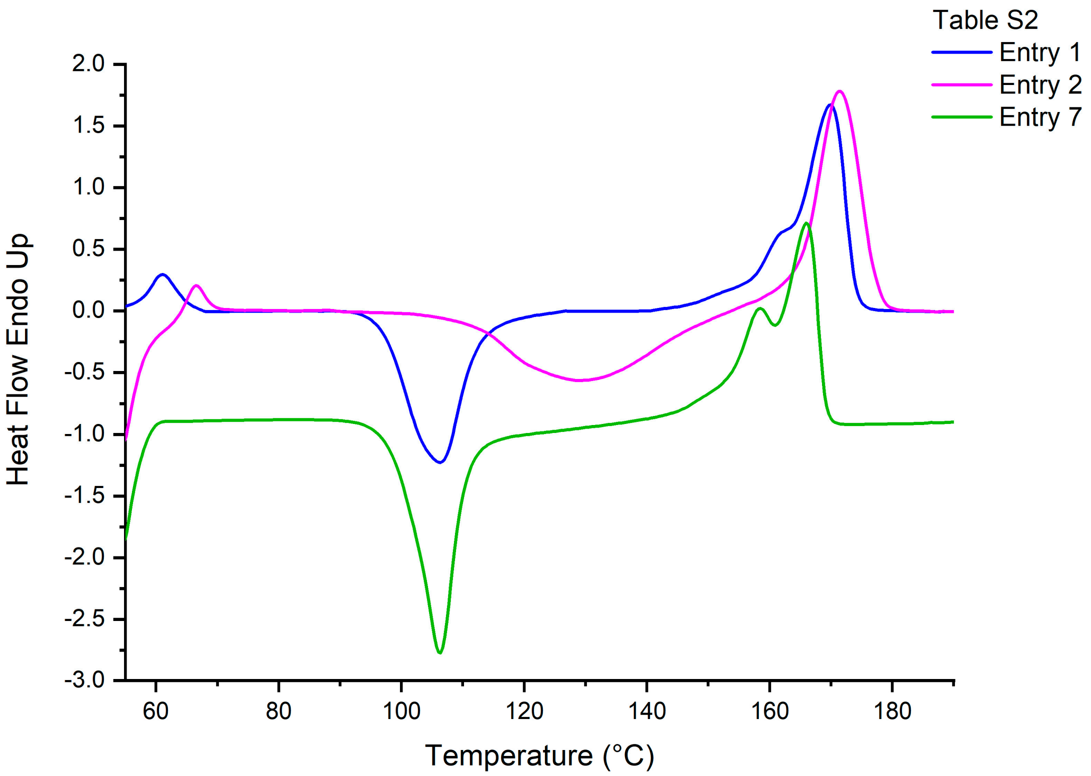

Figure S11. Differential scanning calorimetry (DSC) graphs of PLA. L-lactic 58.4 wt. %, 0.56 wt. % catalyst at 134-137 °C at 92 hours (Table S2, Entry 1) and 120 hours (Table S2, Entry 2). L-lactic 22.8 wt. %, 0.79 wt. % catalyst at 134-137 °C heated for 120 hours and followed by the addition of 0.4 wt. % PDI. The reaction was left stirring at the same temperature for 48 hours (Table S2, Entry 7).

References

(1) Ajioka, Y.; Enomoto, K.; Suzuki K.; Yamaguchi A. The Basic Properties of Poly(lactic Acid) Produced by the Direct Condensation Polymerization of Lactic Acid. *J. Env. Polym. Degrad*. **1995**, *3*, 225–234. https://doi.org/10.1007/BF02068677.

(2) Garlotta, D. A Literature Review of Poly(Lactic Acid). *J. Polym. Environ*. **2001**, 9, 63–84. https://doi.org/10.1023/A:1020200822435.

(3) Woo Lee, H.; Insyani, R.; Prasetyo, D.; Prajitno, H.; Sitompul, J. Molecular Weight and Structural Properties of Biodegradable PLA Synthesized with Different Catalysts by Direct Melt Polycondensation. *J. Eng. Technol. Sci.* **2015**, *47*, 364–373. https://doi.org/10.5614/j.eng.technol.sci.2015.47.4.2.

(4) Liu, C.; Jia, Y.; He, A. Preparation of Higher Molecular Weight Poly (L-Lactic Acid) by Chain Extension. *Int. J. Polym. Sci*. **2013**, Article ID 315917. https://doi.org/10.1155/2013/315917.
